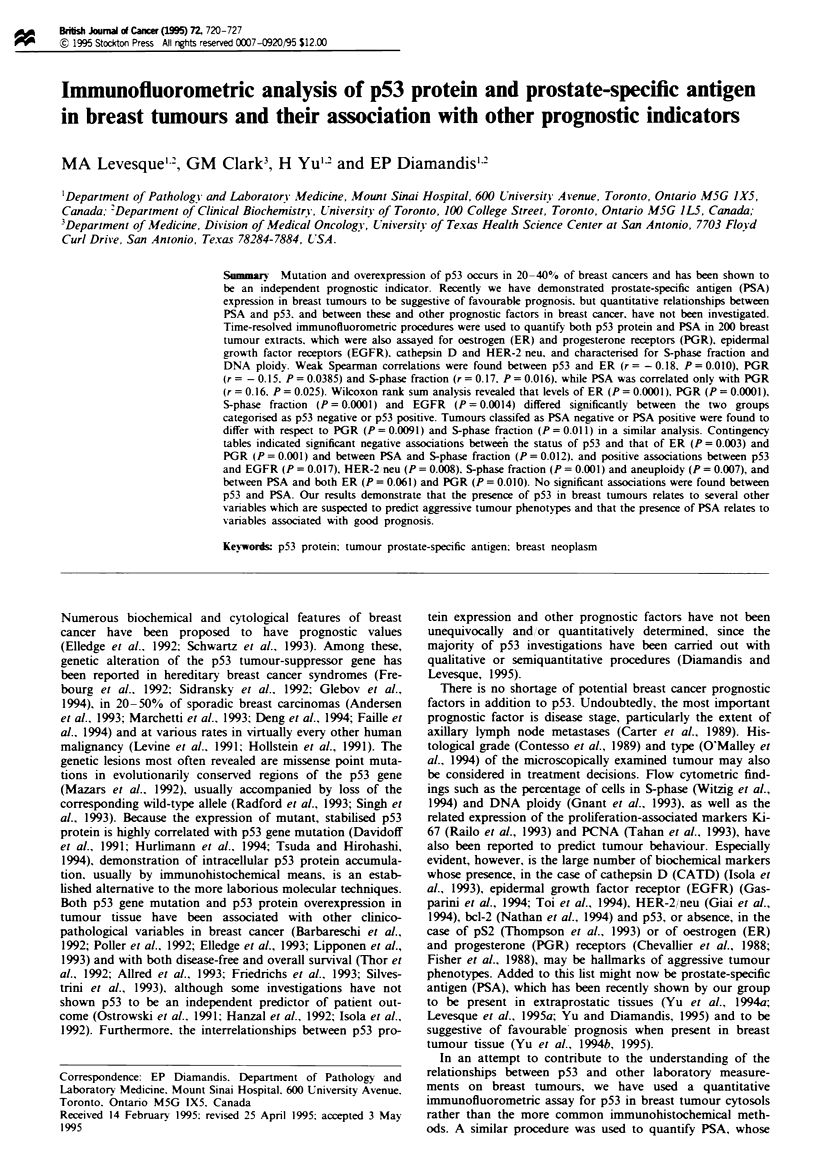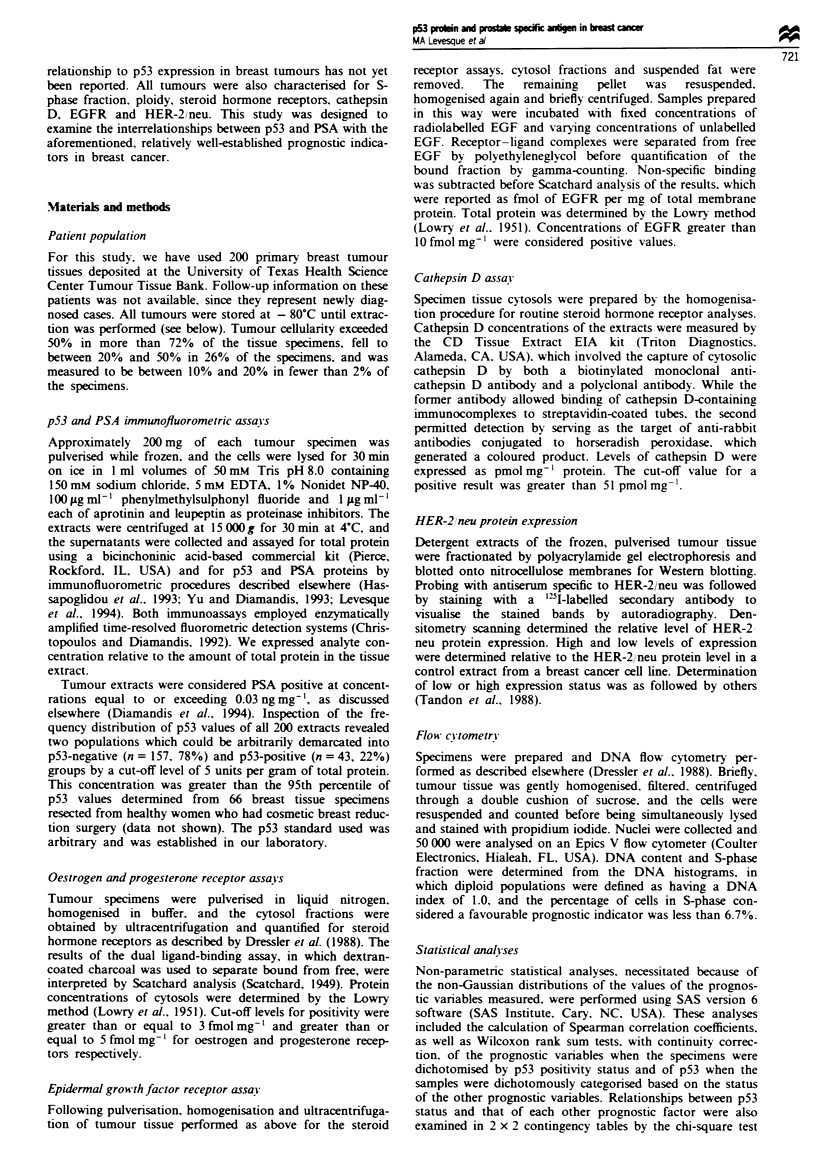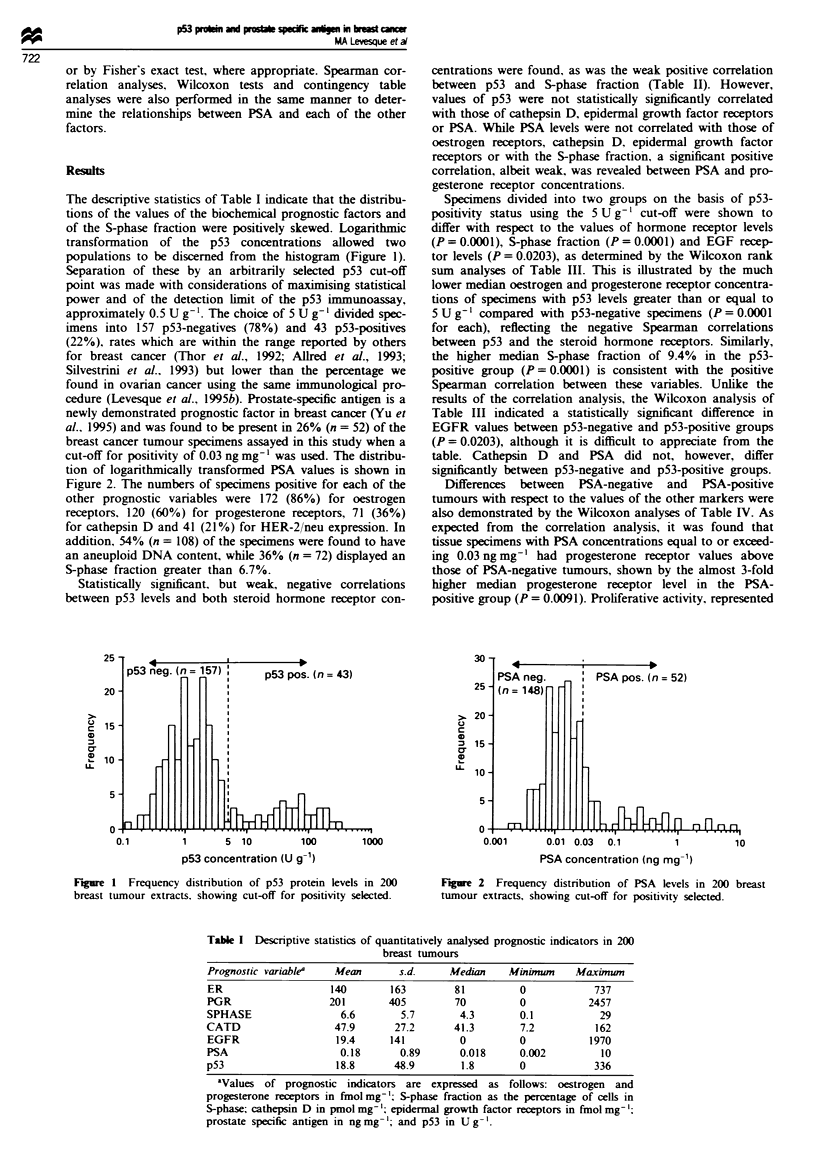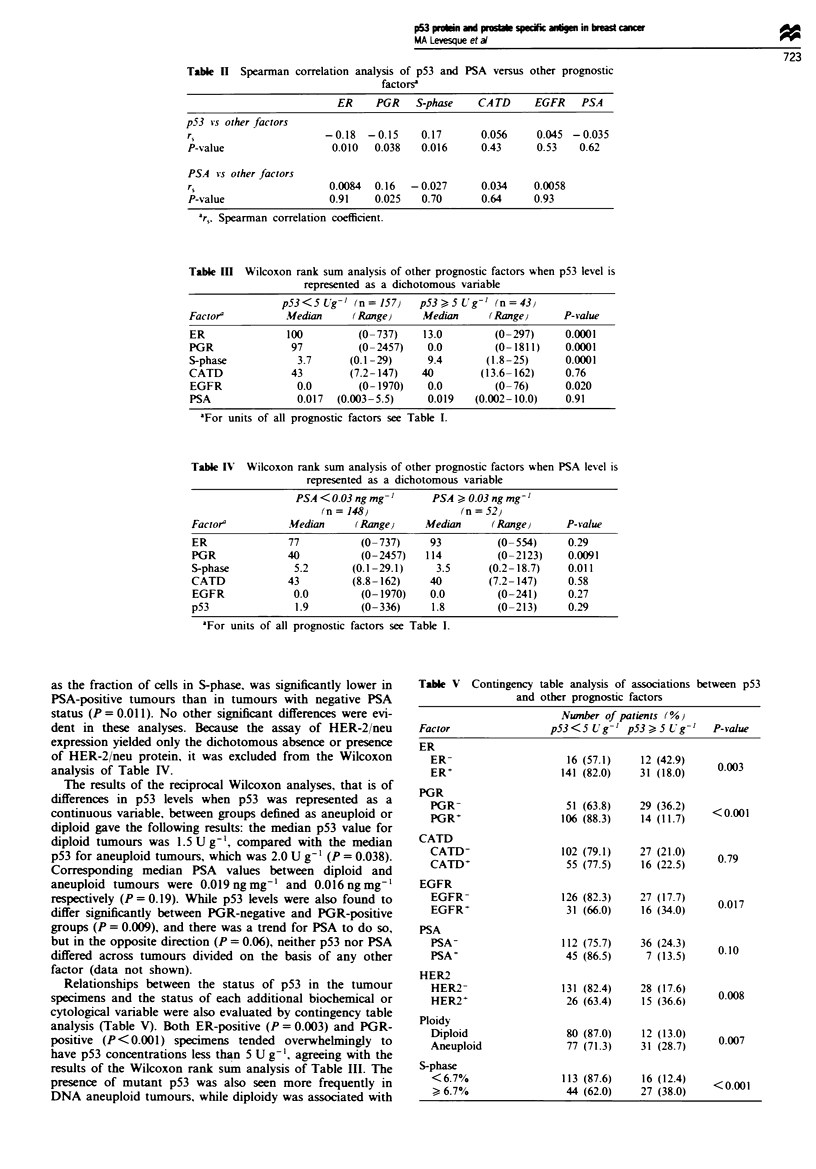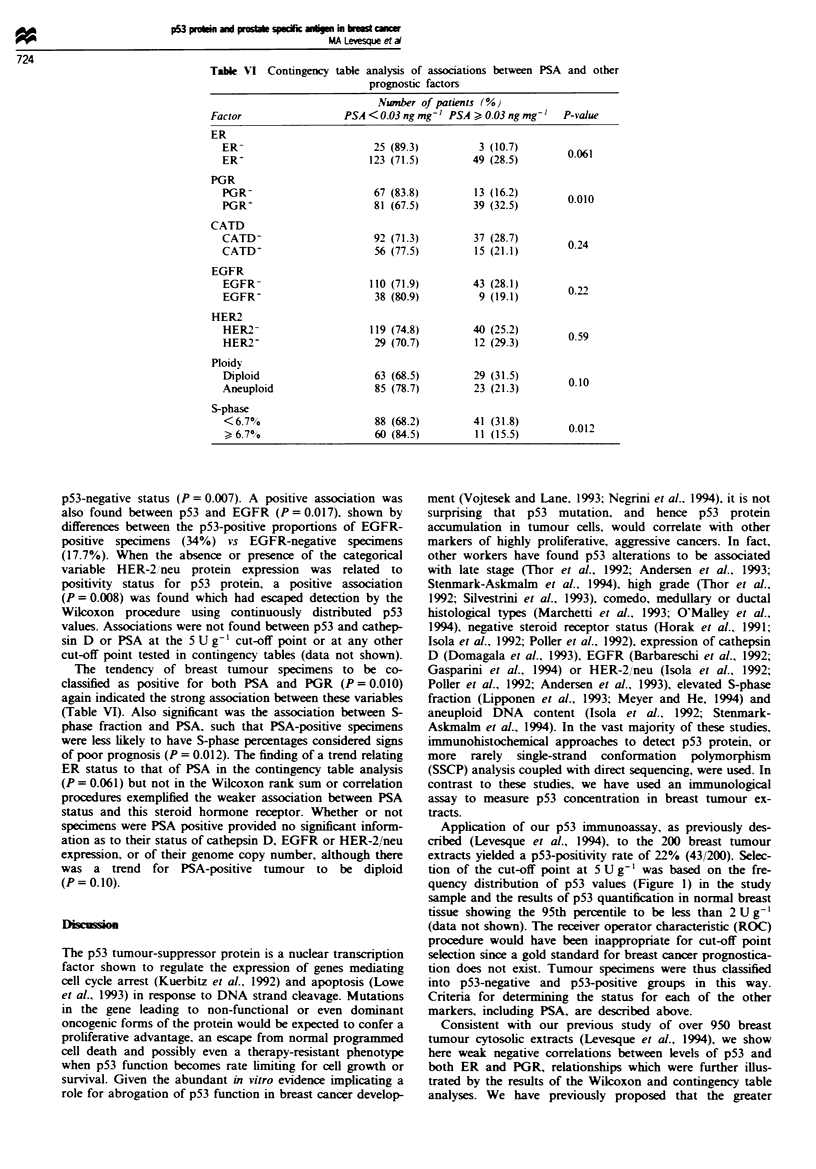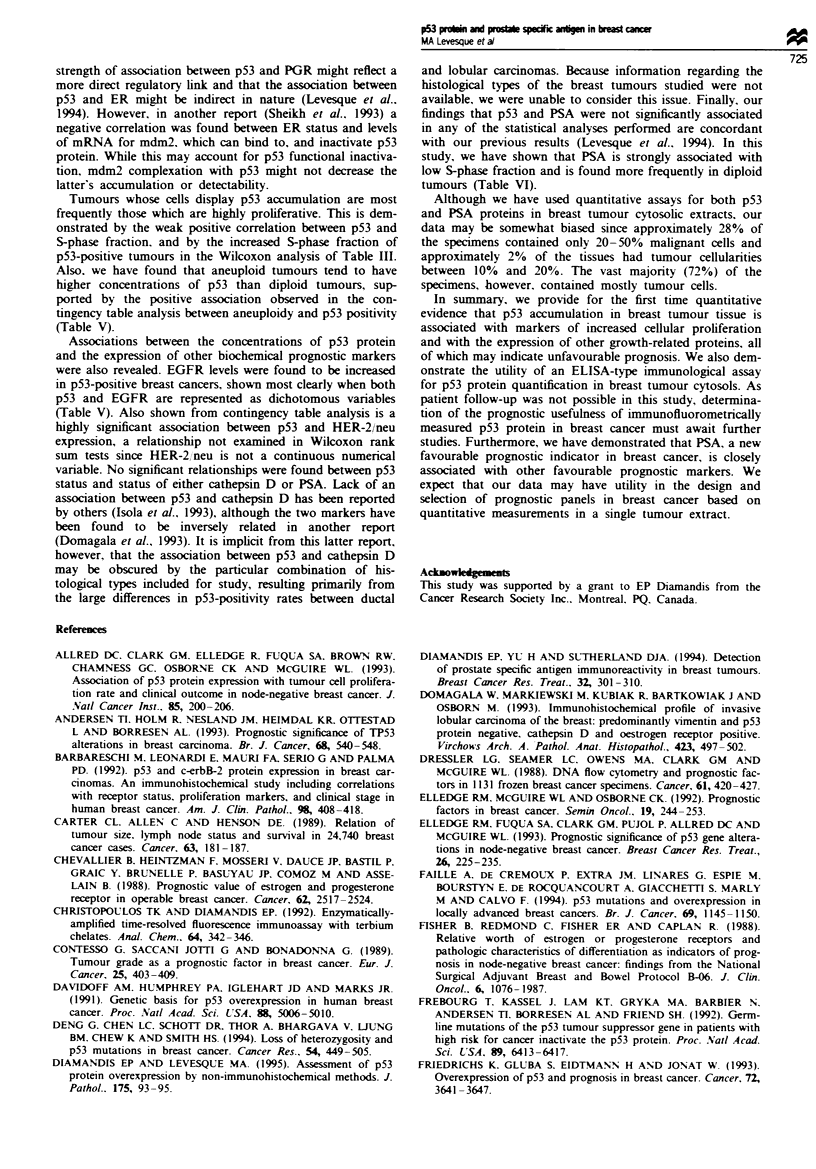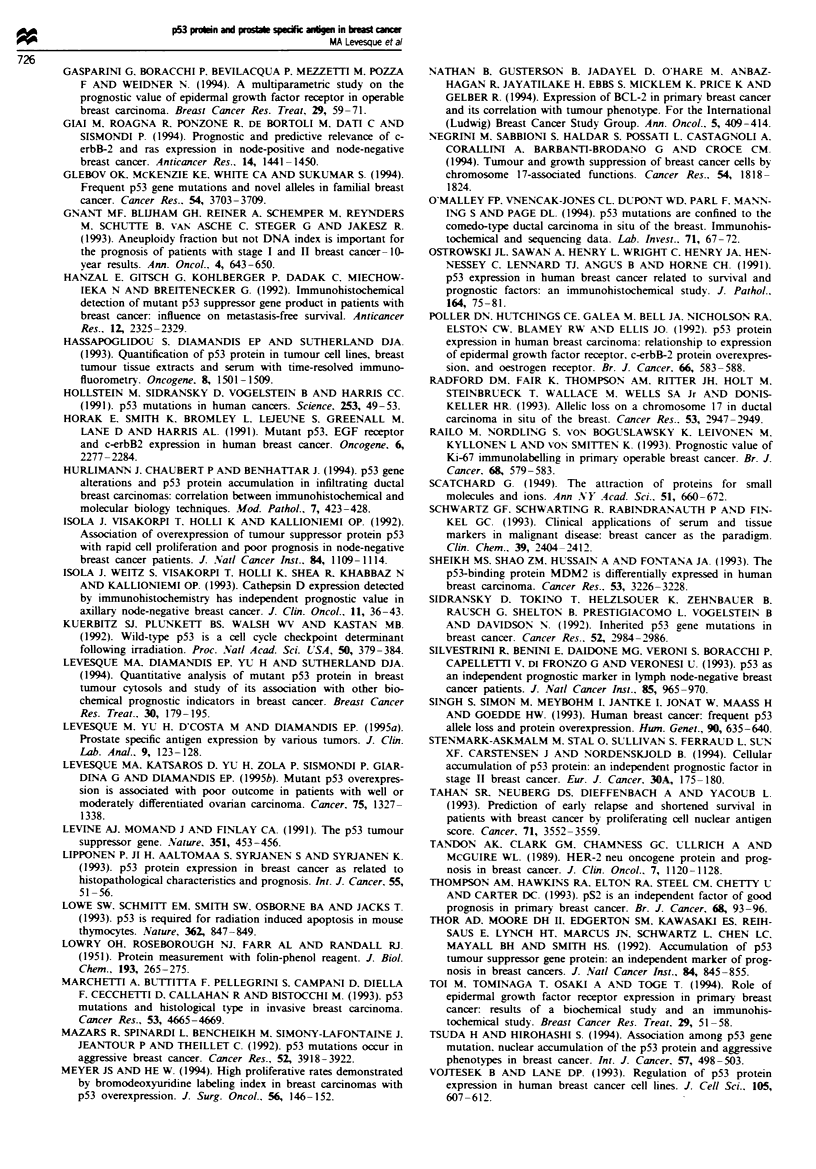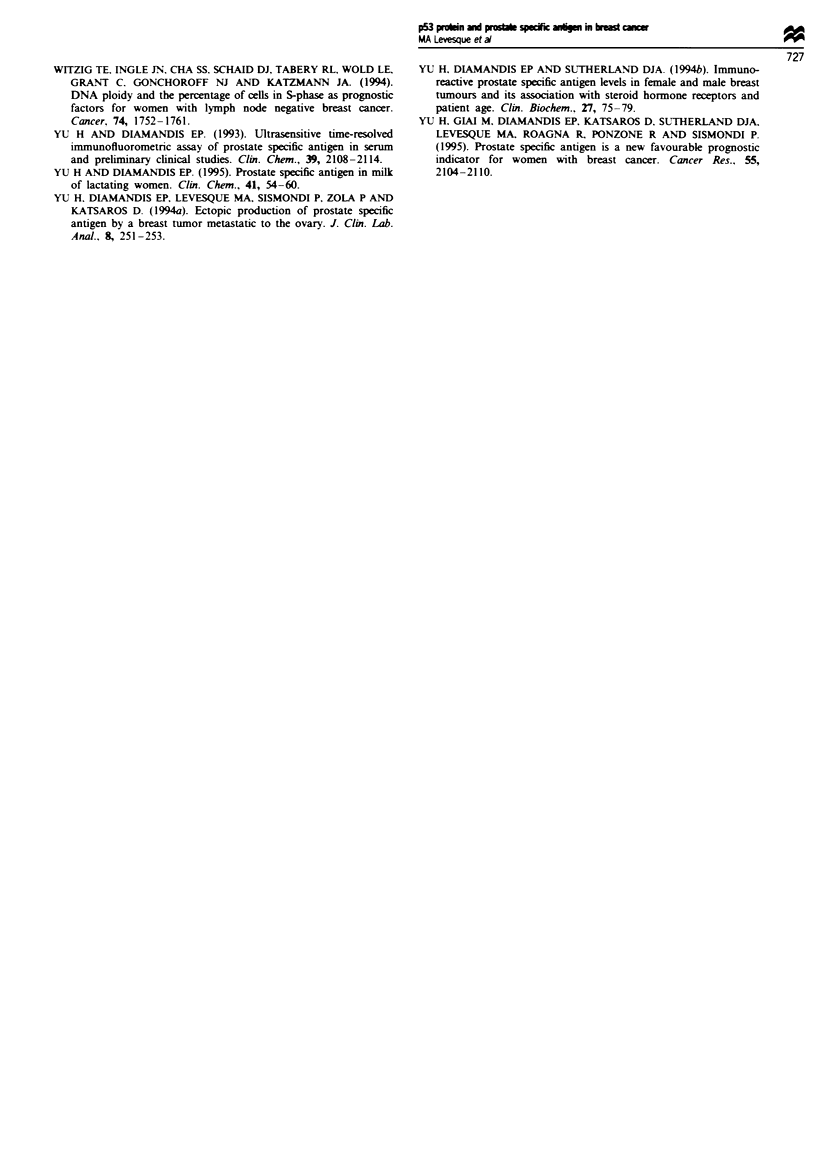# Immunofluorometric analysis of p53 protein and prostate-specific antigen in breast tumours and their association with other prognostic indicators.

**DOI:** 10.1038/bjc.1995.400

**Published:** 1995-09

**Authors:** M. A. Levesque, G. M. Clark, H. Yu, E. P. Diamandis

**Affiliations:** Department of Pathology and Laboratory Medicine, Mount Sinai Hospital, Toronto, Ontario, Canada.

## Abstract

Mutation and overexpression of p53 occurs in 20-40% of breast cancers and has been shown to be an independent prognostic indicator. Recently we have demonstrated prostate-specific antigen (PSA) expression in breast tumours to be suggestive of favourable prognosis, but quantitative relationships between PSA and p53, and between these and other prognostic factors in breast cancer, have not been investigated. Time-resolved immunofluorometric procedures were used to quantify both p53 protein and PSA in 200 breast tumour extracts, which were also assayed for oestrogen (ER) and progesterone receptors (PGR), epidermal growth factor receptors (EGFR), cathepsin D and HER-2/neu, and characterised for S-phase fraction and DNA ploidy. Weak Spearman correlations were found between p53 and ER (r = - 0.18, P = 0.010), PGR (r = - 0.15, P = 0.0385) and S-phase fraction (r = 0.17, P = 0.016), while PSA was correlated only with PGR (r = 0.16, P = 0.025). Wilcoxon rank sum analysis revealed that levels of ER (P = 0.0001), PGR (P = 0.0001), S-phase fraction (P = 0.0001) and EGFR (P = 0.0014) differed significantly between the two groups categorised as p53 negative or p53 positive. Tumours classified as PSA negative or PSA positive were found to differ with respect to PGR (P = 0.0091) and S-phase fraction (P = 0.011) in a similar analysis. Contingency tables indicated significant negative associations between the status of p53 and that of ER (P = 0.003) and PGR (P = 0.001) and between PSA and S-phase fraction (P = 0.012), and positive associations between p53 and EGFR (P = 0.017), HER-2/neu (P = 0.008), S-phase fraction (P = 0.001) and aneuploidy (P = 0.007), and between PSA and both ER (P = 0.061) and PGR (P = 0.010). No significant associations were found between p53 and PSA. Our results demonstrate that the presence of p53 in breast tumours relates to several other variables which are suspected to predict aggressive tumour phenotypes and that the presence of PSA relates to variables associated with good prognosis.


British Journal of Cancer (1995) 72, 720-727

© 1995 Stockton Press All rights reserved 0007-0920/95 $12.00

### Immunofluorometric analysis of p53 protein and prostate-specific antigen in breast tumours and their association with other prognostic indicators

MA Levesque[1,2], GM Clark[3], H Yu[1,2] and EP Diamandis[1,2]

[1]*Department of Pathology and Laboratory Medicine, Mount Sinai Hospital, 600 University Avenue, Toronto, Ontario M5G 1X5, Canada;* [2]*Department of Clinical Biochemistry, University of Toronto, 100 College Street, Toronto, Ontario M5G 1L5, Canada;* [3]*Department of Medicine, Division of Medical Oncology, University of Texas Health Science Center at San Antonio, 7703 Floyd Curl Drive, San Antonio, Texas 78284-7884, USA.*

**Summary** Mutation and overexpression of p53 occurs in 20–40% of breast cancers and has been shown to be an independent prognostic indicator. Recently we have demonstrated prostate-specific antigen (PSA) expression in breast tumours to be suggestive of favourable prognosis, but quantitative relationships between PSA and p53, and between these and other prognostic factors in breast cancer, have not been investigated. Time-resolved immunofluorometric procedures were used to quantify both p53 protein and PSA in 200 breast tumour extracts, which were also assayed for oestrogen (ER) and progesterone receptors (PGR), epidermal growth factor receptors (EGFR), cathepsin D and HER-2/neu, and characterised for S-phase fraction and DNA ploidy. Weak Spearman correlations were found between p53 and ER ($r = -0.18$, $P = 0.010$), PGR ($r = -0.15$, $P = 0.0385$) and S-phase fraction ($r = 0.17$, $P = 0.016$), while PSA was correlated only with PGR ($r = 0.16$, $P = 0.025$). Wilcoxon rank sum analysis revealed that levels of ER ($P = 0.0001$), PGR ($P = 0.0001$), S-phase fraction ($P = 0.0001$) and EGFR ($P = 0.0014$) differed significantly between the two groups categorised as p53 negative or p53 positive. Tumours classifed as PSA negative or PSA positive were found to differ with respect to PGR ($P = 0.0091$) and S-phase fraction ($P = 0.011$) in a similar analysis. Contingency tables indicated significant negative associations between the status of p53 and that of ER ($P = 0.003$) and PGR ($P = 0.001$) and between PSA and S-phase fraction ($P = 0.012$), and positive associations between p53 and EGFR ($P = 0.017$), HER-2/neu ($P = 0.008$), S-phase fraction ($P = 0.001$) and aneuploidy ($P = 0.007$), and between PSA and both ER ($P = 0.061$) and PGR ($P = 0.010$). No significant associations were found between p53 and PSA. Our results demonstrate that the presence of p53 in breast tumours relates to several other variables which are suspected to predict aggressive tumour phenotypes and that the presence of PSA relates to variables associated with good prognosis.

**Keywords:** p53 protein; tumour prostate-specific antigen; breast neoplasm

Numerous biochemical and cytological features of breast cancer have been proposed to have prognostic values (Elledge *et al.*, 1992; Schwartz *et al.*, 1993). Among these, genetic alteration of the p53 tumour-suppressor gene has been reported in hereditary breast cancer syndromes (Freborg *et al.*, 1992; Sidransky *et al.*, 1992; Glebov *et al.*, 1994), in 20–50% of sporadic breast carcinomas (Andersen *et al.*, 1993; Marchetti *et al.*, 1993; Deng *et al.*, 1994; Faille *et al.*, 1994) and at various rates in virtually every other human malignancy (Levine *et al.*, 1991; Hollstein *et al.*, 1991). The genetic lesions most often revealed are missense point mutations in evolutionarily conserved regions of the p53 gene (Mazars *et al.*, 1992), usually accompanied by loss of the corresponding wild-type allele (Radford *et al.*, 1993; Singh *et al.*, 1993). Because the expression of mutant, stabilised p53 protein is highly correlated with p53 gene mutation (Davidoff *et al.*, 1991; Hurlimann *et al.*, 1994; Tsuda and Hirohashi, 1994), demonstration of intracellular p53 protein accumulation, usually by immunohistochemical means, is an established alternative to the more laborious molecular techniques. Both p53 gene mutation and p53 protein overexpression in tumour tissue have been associated with other clinicopathological variables in breast cancer (Barbareschi *et al.*, 1992; Poller *et al.*, 1992; Elledge *et al.*, 1993; Lipponen *et al.*, 1993) and with both disease-free and overall survival (Thor *et al.*, 1992; Allred *et al.*, 1993; Friedrichs *et al.*, 1993; Silvestrini *et al.*, 1993), although some investigations have not shown p53 to be an independent predictor of patient outcome (Ostrowski *et al.*, 1991; Hanzal *et al.*, 1992; Isola *et al.*, 1992). Furthermore, the interrelationships between p53 protein expression and other prognostic factors have not been unequivocally and/or quantitatively determined, since the majority of p53 investigations have been carried out with qualitative or semiquantitative procedures (Diamandis and Levesque, 1995).

There is no shortage of potential breast cancer prognostic factors in addition to p53. Undoubtedly, the most important prognostic factor is disease stage, particularly the extent of axillary lymph node metastases (Carter *et al.*, 1989). Histological grade (Contesso *et al.*, 1989) and type (O'Malley *et al.*, 1994) of the microscopically examined tumour may also be considered in treatment decisions. Flow cytometric findings such as the percentage of cells in S-phase (Witzig *et al.*, 1994) and DNA ploidy (Gnant *et al.*, 1993), as well as the related expression of the proliferation-associated markers Ki-67 (Railo *et al.*, 1993) and PCNA (Tahan *et al.*, 1993), have also been reported to predict tumour behaviour. Especially evident, however, is the large number of biochemical markers whose presence, in the case of cathepsin D (CATD) (Isola *et al.*, 1993), epidermal growth factor receptor (EGFR) (Gasparini *et al.*, 1994; Toi *et al.*, 1994), HER-2/neu (Giai *et al.*, 1994), bcl-2 (Nathan *et al.*, 1994) and p53, or absence, in the case of pS2 (Thompson *et al.*, 1993) or of oestrogen (ER) and progesterone (PGR) receptors (Chevallier *et al.*, 1988; Fisher *et al.*, 1988), may be hallmarks of aggressive tumour phenotypes. Added to this list might now be prostate-specific antigen (PSA), which has been recently shown by our group to be present in extraprostatic tissues (Yu *et al.*, 1994*a*; Levesque *et al.*, 1995*a*; Yu and Diamandis, 1995) and to be suggestive of favourable prognosis when present in breast tumour tissue (Yu *et al.*, 1994*b*, 1995).

In an attempt to contribute to the understanding of the relationships between p53 and other laboratory measurements on breast tumours, we have used a quantitative immunofluorometric assay for p53 in breast tumour cytosols rather than the more common immunohistochemical methods. A similar procedure was used to quantify PSA, whose

Correspondence: EP Diamandis, Department of Pathology and Laboratory Medicine, Mount Sinai Hospital, 600 University Avenue, Toronto, Ontario M5G 1X5, Canada
Received 14 February 1995; revised 25 April 1995; accepted 3 May 1995

relationship to p53 expression in breast tumours has not yet been reported. All tumours were also characterised for S-phase fraction, ploidy, steroid hormone receptors, cathepsin D, EGFR and HER-2/neu. This study was designed to examine the interrelationships between p53 and PSA with the aforementioned, relatively well-established prognostic indicators in breast cancer.

### Materials and methods

### *Patient population*

For this study, we have used 200 primary breast tumour tissues deposited at the University of Texas Health Science Center Tumour Tissue Bank. Follow-up information on these patients was not available, since they represent newly diagnosed cases. All tumours were stored at − 80°C until extraction was performed (see below). Tumour cellularity exceeded 50% in more than 72% of the tissue specimens, fell to between 20% and 50% in 26% of the specimens, and was measured to be between 10% and 20% in fewer than 2% of the specimens.

### *p53 and PSA immunofluorometric assays*

Approximately 200 mg of each tumour specimen was pulverised while frozen, and the cells were lysed for 30 min on ice in 1 ml volumes of 50 mM Tris pH 8.0 containing 150 mM sodium chloride, 5 mM EDTA, 1% Nonidet NP-40, 100 $\mu g\ ml^{-1}$ phenylmethylsulphonyl fluoride and 1 $\mu g\ ml^{-1}$ each of aprotinin and leupeptin as proteinase inhibitors. The extracts were centrifuged at 15 000 *g* for 30 min at 4°C, and the supernatants were collected and assayed for total protein using a bicinchoninic acid-based commercial kit (Pierce, Rockford, IL, USA) and for p53 and PSA proteins by immunofluorometric procedures described elsewhere (Hassapoglidou *et al*., 1993; Yu and Diamandis, 1993; Levesque *et al*., 1994). Both immunoassays employed enzymatically amplified time-resolved fluorometric detection systems (Christopoulos and Diamandis, 1992). We expressed analyte concentration relative to the amount of total protein in the tissue extract.

Tumour extracts were considered PSA positive at concentrations equal to or exceeding 0.03 $ng\ mg^{-1}$, as discussed elsewhere (Diamandis *et al*., 1994). Inspection of the frequency distribution of p53 values of all 200 extracts revealed two populations which could be arbitrarily demarcated into p53-negative ($n = 157$, 78%) and p53-positive ($n = 43$, 22%) groups by a cut-off level of 5 units per gram of total protein. This concentration was greater than the 95th percentile of p53 values determined from 66 breast tissue specimens resected from healthy women who had cosmetic breast reduction surgery (data not shown). The p53 standard used was arbitrary and was established in our laboratory.

### *Oestrogen and progesterone receptor assays*

Tumour specimens were pulverised in liquid nitrogen, homogenised in buffer, and the cytosol fractions were obtained by ultracentrifugation and quantified for steroid hormone receptors as described by Dressler *et al*. (1988). The results of the dual ligand-binding assay, in which dextran-coated charcoal was used to separate bound from free, were interpreted by Scatchard analysis (Scatchard, 1949). Protein concentrations of cytosols were determined by the Lowry method (Lowry *et al*., 1951). Cut-off levels for positivity were greater than or equal to 3 $fmol\ mg^{-1}$ and greater than or equal to 5 $fmol\ mg^{-1}$ for oestrogen and progesterone receptors respectively.

### *Epidermal growth factor receptor assay*

Following pulverisation, homogenisation and ultracentrifugation of tumour tissue performed as above for the steroid receptor assays, cytosol fractions and suspended fat were removed. The remaining pellet was resuspended, homogenised again and briefly centrifuged. Samples prepared in this way were incubated with fixed concentrations of radiolabelled EGF and varying concentrations of unlabelled EGF. Receptor–ligand complexes were separated from free EGF by polyethyleneglycol before quantification of the bound fraction by gamma-counting. Non-specific binding was subtracted before Scatchard analysis of the results, which were reported as fmol of EGFR per mg of total membrane protein. Total protein was determined by the Lowry method (Lowry *et al*., 1951). Concentrations of EGFR greater than 10 $fmol\ mg^{-1}$ were considered positive values.

### *Cathepsin D assay*

Specimen tissue cytosols were prepared by the homogenisation procedure for routine steroid hormone receptor analyses. Cathepsin D concentrations of the extracts were measured by the CD Tissue Extract EIA kit (Triton Diagnostics, Alameda, CA, USA), which involved the capture of cytosolic cathepsin D by both a biotinylated monoclonal anti-cathepsin D antibody and a polyclonal antibody. While the former antibody allowed binding of cathepsin D-containing immunocomplexes to streptavidin-coated tubes, the second permitted detection by serving as the target of anti-rabbit antibodies conjugated to horseradish peroxidase, which generated a coloured product. Levels of cathepsin D were expressed as $pmol\ mg^{-1}$ protein. The cut-off value for a positive result was greater than 51 $pmol\ mg^{-1}$.

### *HER-2/neu protein expression*

Detergent extracts of the frozen, pulverised tumour tissue were fractionated by polyacrylamide gel electrophoresis and blotted onto nitrocellulose membranes for Western blotting. Probing with antiserum specific to HER-2/neu was followed by staining with a $^{125}I$-labelled secondary antibody to visualise the stained bands by autoradiography. Densitometry scanning determined the relative level of HER-2/neu protein expression. High and low levels of expression were determined relative to the HER-2/neu protein level in a control extract from a breast cancer cell line. Determination of low or high expression status was as followed by others (Tandon *et al*., 1988).

### *Flow cytometry*

Specimens were prepared and DNA flow cytometry performed as described elsewhere (Dressler *et al*., 1988). Briefly, tumour tissue was gently homogenised, filtered, centrifuged through a double cushion of sucrose, and the cells were resuspended and counted before being simultaneously lysed and stained with propidium iodide. Nuclei were collected and 50 000 were analysed on an Epics V flow cytometer (Coulter Electronics, Hialeah, FL, USA). DNA content and S-phase fraction were determined from the DNA histograms, in which diploid populations were defined as having a DNA index of 1.0, and the percentage of cells in S-phase considered a favourable prognostic indicator was less than 6.7%.

### *Statistical analyses*

Non-parametric statistical analyses, necessitated because of the non-Gaussian distributions of the values of the prognostic variables measured, were performed using SAS version 6 software (SAS Institute, Cary, NC, USA). These analyses included the calculation of Spearman correlation coefficients, as well as Wilcoxon rank sum tests, with continuity correction, of the prognostic variables when the specimens were dichotomised by p53 positivity status and of p53 when the samples were dichotomously categorised based on the status of the other prognostic variables. Relationships between p53 status and that of each other prognostic factor were also examined in $2 \times 2$ contingency tables by the chi-square test or by Fisher's exact test, where appropriate. Spearman correlation analyses, Wilcoxon tests and contingency table analyses were also performed in the same manner to determine the relationships between PSA and each of the other factors.

### Results

The descriptive statistics of Table I indicate that the distributions of the values of the biochemical prognostic factors and of the S-phase fraction were positively skewed. Logarithmic transformation of the p53 concentrations allowed two populations to be discerned from the histogram (Figure 1). Separation of these by an arbitrarily selected p53 cut-off point was made with considerations of maximising statistical power and of the detection limit of the p53 immunoassay, approximately 0.5 U $g^{-1}$. The choice of 5 U $g^{-1}$ divided specimens into 157 p53-negatives (78%) and 43 p53-positives (22%), rates which are within the range reported by others for breast cancer (Thor *et al.*, 1992; Allred *et al.*, 1993; Silvestrini *et al.*, 1993) but lower than the percentage we found in ovarian cancer using the same immunological procedure (Levesque *et al.*, 1995*b*). Prostate-specific antigen is a newly demonstrated prognostic factor in breast cancer (Yu *et al.*, 1995) and was found to be present in 26% ($n$ = 52) of the breast cancer tumour specimens assayed in this study when a cut-off for positivity of 0.03 ng $mg^{-1}$ was used. The distribution of logarithmically transformed PSA values is shown in Figure 2. The numbers of specimens positive for each of the other prognostic variables were 172 (86%) for oestrogen receptors, 120 (60%) for progesterone receptors, 71 (36%) for cathepsin D and 41 (21%) for HER-2/neu expression. In addition, 54% ($n$ = 108) of the specimens were found to have an aneuploid DNA content, while 36% ($n$ = 72) displayed an S-phase fraction greater than 6.7%.

Statistically significant, but weak, negative correlations between p53 levels and both steroid hormone receptor concentrations were found, as was the weak positive correlation between p53 and S-phase fraction (Table II). However, values of p53 were not statistically significantly correlated with those of cathepsin D, epidermal growth factor receptors or PSA. While PSA levels were not correlated with those of oestrogen receptors, cathepsin D, epidermal growth factor receptors or with the S-phase fraction, a significant positive correlation, albeit weak, was revealed between PSA and progesterone receptor concentrations.

Specimens divided into two groups on the basis of p53-positivity status using the 5 U $g^{-1}$ cut-off were shown to differ with respect to the values of hormone receptor levels ($P$ = 0.0001), S-phase fraction ($P$ = 0.0001) and EGF receptor levels ($P$ = 0.0203), as determined by the Wilcoxon rank sum analyses of Table III. This is illustrated by the much lower median oestrogen and progesterone receptor concentrations of specimens with p53 levels greater than or equal to 5 U $g^{-1}$ compared with p53-negative specimens ($P$ = 0.0001 for each), reflecting the negative Spearman correlations between p53 and the steroid hormone receptors. Similarly, the higher median S-phase fraction of 9.4% in the p53-positive group ($P$ = 0.0001) is consistent with the positive Spearman correlation between these variables. Unlike the results of the correlation analysis, the Wilcoxon analysis of Table III indicated a statistically significant difference in EGFR values between p53-negative and p53-positive groups ($P$ = 0.0203), although it is difficult to appreciate from the table. Cathepsin D and PSA did not, however, differ significantly between p53-negative and p53-positive groups.

Differences between PSA-negative and PSA-positive tumours with respect to the values of the other markers were also demonstrated by the Wilcoxon analyses of Table IV. As expected from the correlation analysis, it was found that tissue specimens with PSA concentrations equal to or exceeding 0.03 ng $mg^{-1}$ had progesterone receptor values above those of PSA-negative tumours, shown by the almost 3-fold higher median progesterone receptor level in the PSA-positive group ($P$ = 0.0091). Proliferative activity, represented

**Figure 1** Frequency distribution of p53 protein levels in 200 breast tumour extracts, showing cut-off for positivity selected.

**Figure 2** Frequency distribution of PSA levels in 200 breast tumour extracts, showing cut-off for positivity selected.

**Table I** Descriptive statistics of quantitatively analysed prognostic indicators in 200 breast tumours

| *Prognostic variable*[a] | *Mean* | *s.d.* | *Median* | *Minimum* | *Maximum* |
|---|---|---|---|---|---|
| ER | 140 | 163 | 81 | 0 | 737 |
| PGR | 201 | 405 | 70 | 0 | 2457 |
| SPHASE | 6.6 | 5.7 | 4.3 | 0.1 | 29 |
| CATD | 47.9 | 27.2 | 41.3 | 7.2 | 162 |
| EGFR | 19.4 | 141 | 0 | 0 | 1970 |
| PSA | 0.18 | 0.89 | 0.018 | 0.002 | 10 |
| p53 | 18.8 | 48.9 | 1.8 | 0 | 336 |

[a]Values of prognostic indicators are expressed as follows: oestrogen and progesterone receptors in fmol $mg^{-1}$; S-phase fraction as the percentage of cells in S-phase; cathepsin D in pmol $mg^{-1}$; epidermal growth factor receptors in fmol $mg^{-1}$; prostate specific antigen in ng $mg^{-1}$; and p53 in U $g^{-1}$.

**Table II** Spearman correlation analysis of p53 and PSA versus other prognostic factors[a]

| | *ER* | *PGR* | *S-phase* | *CATD* | *EGFR* | *PSA* |
|---|---|---|---|---|---|---|
| *p53 vs other factors* | | | | | | |
| $r_s$ | −0.18 | −0.15 | 0.17 | 0.056 | 0.045 | −0.035 |
| *P*-value | 0.010 | 0.038 | 0.016 | 0.43 | 0.53 | 0.62 |
| *PSA vs other factors* | | | | | | |
| $r_s$ | 0.0084 | 0.16 | −0.027 | 0.034 | 0.0058 | |
| *P*-value | 0.91 | 0.025 | 0.70 | 0.64 | 0.93 | |

[a]$r_s$, Spearman correlation coefficient.

**Table III** Wilcoxon rank sum analysis of other prognostic factors when p53 level is represented as a dichotomous variable

| | *p53<5 Ug⁻¹ (n = 157)* | | *p53≥5 U g⁻¹ (n = 43)* | | |
|---|---|---|---|---|---|
| *Factor*[a] | *Median* | *(Range)* | *Median* | *(Range)* | *P-value* |
| ER | 100 | (0–737) | 13.0 | (0–297) | 0.0001 |
| PGR | 97 | (0–2457) | 0.0 | (0–1811) | 0.0001 |
| S-phase | 3.7 | (0.1–29) | 9.4 | (1.8–25) | 0.0001 |
| CATD | 43 | (7.2–147) | 40 | (13.6–162) | 0.76 |
| EGFR | 0.0 | (0–1970) | 0.0 | (0–76) | 0.020 |
| PSA | 0.017 | (0.003–5.5) | 0.019 | (0.002–10.0) | 0.91 |

[a]For units of all prognostic factors see Table I.

**Table IV** Wilcoxon rank sum analysis of other prognostic factors when PSA level is represented as a dichotomous variable

| | *PSA<0.03 ng mg⁻¹ (n = 148)* | | *PSA ≥ 0.03 ng mg⁻¹ (n = 52)* | | |
|---|---|---|---|---|---|
| *Factor*[a] | *Median* | *(Range)* | *Median* | *(Range)* | *P-value* |
| ER | 77 | (0–737) | 93 | (0–554) | 0.29 |
| PGR | 40 | (0–2457) | 114 | (0–2123) | 0.0091 |
| S-phase | 5.2 | (0.1–29.1) | 3.5 | (0.2–18.7) | 0.011 |
| CATD | 43 | (8.8–162) | 40 | (7.2–147) | 0.58 |
| EGFR | 0.0 | (0–1970) | 0.0 | (0–241) | 0.27 |
| p53 | 1.9 | (0–336) | 1.8 | (0–213) | 0.29 |

[a]For units of all prognostic factors see Table I.

as the fraction of cells in S-phase, was significantly lower in PSA-positive tumours than in tumours with negative PSA status ($P = 0.011$). No other significant differences were evident in these analyses. Because the assay of HER-2/neu expression yielded only the dichotomous absence or presence of HER-2/neu protein, it was excluded from the Wilcoxon analysis of Table IV.

The results of the reciprocal Wilcoxon analyses, that is of differences in p53 levels when p53 was represented as a continuous variable, between groups defined as aneuploid or diploid gave the following results: the median p53 value for diploid tumours was 1.5 U $g^{-1}$, compared with the median p53 for aneuploid tumours, which was 2.0 U $g^{-1}$ ($P = 0.038$). Corresponding median PSA values between diploid and aneuploid tumours were 0.019 ng $mg^{-1}$ and 0.016 ng $mg^{-1}$ respectively ($P = 0.19$). While p53 levels were also found to differ significantly between PGR-negative and PGR-positive groups ($P = 0.009$), and there was a trend for PSA to do so, but in the opposite direction ($P = 0.06$), neither p53 nor PSA differed across tumours divided on the basis of any other factor (data not shown).

Relationships between the status of p53 in the tumour specimens and the status of each additional biochemical or cytological variable were also evaluated by contingency table analysis (Table V). Both ER-positive ($P = 0.003$) and PGR-positive ($P < 0.001$) specimens tended overwhelmingly to have p53 concentrations less than 5 U $g^{-1}$, agreeing with the results of the Wilcoxon rank sum analysis of Table III. The presence of mutant p53 was also seen more frequently in DNA aneuploid tumours, while diploidy was associated with

**Table V** Contingency table analysis of associations between p53 and other prognostic factors

| | *Number of patients (%)* | | |
|---|---|---|---|
| *Factor* | *p53<5 U g⁻¹* | *p53≥5 U g⁻¹* | *P-value* |
| ER | | | |
| ER⁻ | 16 (57.1) | 12 (42.9) | 0.003 |
| ER⁺ | 141 (82.0) | 31 (18.0) | |
| PGR | | | |
| PGR⁻ | 51 (63.8) | 29 (36.2) | <0.001 |
| PGR⁺ | 106 (88.3) | 14 (11.7) | |
| CATD | | | |
| CATD⁻ | 102 (79.1) | 27 (21.0) | 0.79 |
| CATD⁺ | 55 (77.5) | 16 (22.5) | |
| EGFR | | | |
| EGFR⁻ | 126 (82.3) | 27 (17.7) | 0.017 |
| EGFR⁺ | 31 (66.0) | 16 (34.0) | |
| PSA | | | |
| PSA⁻ | 112 (75.7) | 36 (24.3) | 0.10 |
| PSA⁺ | 45 (86.5) | 7 (13.5) | |
| HER2 | | | |
| HER2⁻ | 131 (82.4) | 28 (17.6) | 0.008 |
| HER2⁺ | 26 (63.4) | 15 (36.6) | |
| Ploidy | | | |
| Diploid | 80 (87.0) | 12 (13.0) | 0.007 |
| Aneuploid | 77 (71.3) | 31 (28.7) | |
| S-phase | | | |
| <6.7% | 113 (87.6) | 16 (12.4) | <0.001 |
| ≥6.7% | 44 (62.0) | 27 (38.0) | |

**Table VI** Contingency table analysis of associations between PSA and other prognostic factors

| Factor | Number of patients (%) PSA $<0.03$ ng mg$^{-1}$ | PSA $\geqslant 0.03$ ng mg$^{-1}$ | P-value |
|---|---|---|---|
| ER | | | |
| $ER^-$ | 25 (89.3) | 3 (10.7) | 0.061 |
| $ER^+$ | 123 (71.5) | 49 (28.5) | |
| PGR | | | |
| $PGR^-$ | 67 (83.8) | 13 (16.2) | 0.010 |
| $PGR^+$ | 81 (67.5) | 39 (32.5) | |
| CATD | | | |
| $CATD^-$ | 92 (71.3) | 37 (28.7) | 0.24 |
| $CATD^+$ | 56 (77.5) | 15 (21.1) | |
| EGFR | | | |
| $EGFR^-$ | 110 (71.9) | 43 (28.1) | 0.22 |
| $EGFR^+$ | 38 (80.9) | 9 (19.1) | |
| HER2 | | | |
| $HER2^-$ | 119 (74.8) | 40 (25.2) | 0.59 |
| $HER2^+$ | 29 (70.7) | 12 (29.3) | |
| Ploidy | | | |
| Diploid | 63 (68.5) | 29 (31.5) | 0.10 |
| Aneuploid | 85 (78.7) | 23 (21.3) | |
| S-phase | | | |
| $<6.7\%$ | 88 (68.2) | 41 (31.8) | 0.012 |
| $\geqslant 6.7\%$ | 60 (84.5) | 11 (15.5) | |

p53-negative status ($P = 0.007$). A positive association was also found between p53 and EGFR ($P = 0.017$), shown by differences between the p53-positive proportions of EGFR-positive specimens (34%) *vs* EGFR-negative specimens (17.7%). When the absence or presence of the categorical variable HER-2/neu protein expression was related to positivity status for p53 protein, a positive association ($P = 0.008$) was found which had escaped detection by the Wilcoxon procedure using continuously distributed p53 values. Associations were not found between p53 and cathepsin D or PSA at the 5 U g$^{-1}$ cut-off point or at any other cut-off point tested in contingency tables (data not shown).

The tendency of breast tumour specimens to be co-classified as positive for both PSA and PGR ($P = 0.010$) again indicated the strong association between these variables (Table VI). Also significant was the association between S-phase fraction and PSA, such that PSA-positive specimens were less likely to have S-phase percentages considered signs of poor prognosis ($P = 0.012$). The finding of a trend relating ER status to that of PSA in the contingency table analysis ($P = 0.061$) but not in the Wilcoxon rank sum or correlation procedures exemplified the weaker association between PSA status and this steroid hormone receptor. Whether or not specimens were PSA positive provided no significant information as to their status of cathepsin D, EGFR or HER-2/neu expression, or of their genome copy number, although there was a trend for PSA-positive tumour to be diploid ($P = 0.10$).

### Discussion

The p53 tumour-suppressor protein is a nuclear transcription factor shown to regulate the expression of genes mediating cell cycle arrest (Kuerbitz *et al.*, 1992) and apoptosis (Lowe *et al.*, 1993) in response to DNA strand cleavage. Mutations in the gene leading to non-functional or even dominant oncogenic forms of the protein would be expected to confer a proliferative advantage, an escape from normal programmed cell death and possibly even a therapy-resistant phenotype when p53 function becomes rate limiting for cell growth or survival. Given the abundant *in vitro* evidence implicating a role for abrogation of p53 function in breast cancer development (Vojtesek and Lane, 1993; Negrini *et al.*, 1994), it is not surprising that p53 mutation, and hence p53 protein accumulation in tumour cells, would correlate with other markers of highly proliferative, aggressive cancers. In fact, other workers have found p53 alterations to be associated with late stage (Thor *et al.*, 1992; Andersen *et al.*, 1993; Stenmark-Askmalm *et al.*, 1994), high grade (Thor *et al.*, 1992; Silvestrini *et al.*, 1993), comedo, medullary or ductal histological types (Marchetti *et al.*, 1993; O'Malley *et al.*, 1994), negative steroid receptor status (Horak *et al.*, 1991; Isola *et al.*, 1992; Poller *et al.*, 1992), expression of cathepsin D (Domagala *et al.*, 1993), EGFR (Barbareschi *et al.*, 1992; Gasparini *et al.*, 1994) or HER-2/neu (Isola *et al.*, 1992; Poller *et al.*, 1992; Andersen *et al.*, 1993), elevated S-phase fraction (Lipponen *et al.*, 1993; Meyer and He, 1994) and aneuploid DNA content (Isola *et al.*, 1992; Stenmark-Askmalm *et al.*, 1994). In the vast majority of these studies, immunohistochemical approaches to detect p53 protein, or more rarely single-strand conformation polymorphism (SSCP) analysis coupled with direct sequencing, were used. In contrast to these studies, we have used an immunological assay to measure p53 concentration in breast tumour extracts.

Application of our p53 immunoassay, as previously described (Levesque *et al.*, 1994), to the 200 breast tumour extracts yielded a p53-positivity rate of 22% (43/200). Selection of the cut-off point at 5 U g$^{-1}$ was based on the frequency distribution of p53 values (Figure 1) in the study sample and the results of p53 quantification in normal breast tissue showing the 95th percentile to be less than 2 U g$^{-1}$ (data not shown). The receiver operator characteristic (ROC) procedure would have been inappropriate for cut-off point selection since a gold standard for breast cancer prognostication does not exist. Tumour specimens were thus classified into p53-negative and p53-positive groups in this way. Criteria for determining the status for each of the other markers, including PSA, are described above.

Consistent with our previous study of over 950 breast tumour cytosolic extracts (Levesque *et al.*, 1994), we show here weak negative correlations between levels of p53 and both ER and PGR, relationships which were further illustrated by the results of the Wilcoxon and contingency table analyses. We have previously proposed that the greater strength of association between p53 and PGR might reflect a more direct regulatory link and that the association between p53 and ER might be indirect in nature (Levesque *et al.*, 1994). However, in another report (Sheikh *et al.*, 1993) a negative correlation was found between ER status and levels of mRNA for mdm2, which can bind to, and inactivate p53 protein. While this may account for p53 functional inactivation, mdm2 complexation with p53 might not decrease the latter's accumulation or detectability.

Tumours whose cells display p53 accumulation are most frequently those which are highly proliferative. This is demonstrated by the weak positive correlation between p53 and S-phase fraction, and by the increased S-phase fraction of p53-positive tumours in the Wilcoxon analysis of Table III. Also, we have found that aneuploid tumours tend to have higher concentrations of p53 than diploid tumours, supported by the positive association observed in the contingency table analysis between aneuploidy and p53 positivity (Table V).

Associations between the concentrations of p53 protein and the expression of other biochemical prognostic markers were also revealed. EGFR levels were found to be increased in p53-positive breast cancers, shown most clearly when both p53 and EGFR are represented as dichotomous variables (Table V). Also shown from contingency table analysis is a highly significant association between p53 and HER-2/neu expression, a relationship not examined in Wilcoxon rank sum tests since HER-2/neu is not a continuous numerical variable. No significant relationships were found between p53 status and status of either cathepsin D or PSA. Lack of an association between p53 and cathepsin D has been reported by others (Isola *et al.*, 1993), although the two markers have been found to be inversely related in another report (Domagala *et al.*, 1993). It is implicit from this latter report, however, that the association between p53 and cathepsin D may be obscured by the particular combination of histological types included for study, resulting primarily from the large differences in p53-positivity rates between ductal and lobular carcinomas. Because information regarding the histological types of the breast tumours studied were not available, we were unable to consider this issue. Finally, our findings that p53 and PSA were not significantly associated in any of the statistical analyses performed are concordant with our previous results (Levesque *et al.*, 1994). In this study, we have shown that PSA is strongly associated with low S-phase fraction and is found more frequently in diploid tumours (Table VI).

Although we have used quantitative assays for both p53 and PSA proteins in breast tumour cytosolic extracts, our data may be somewhat biased since approximately 28% of the specimens contained only 20–50% malignant cells and approximately 2% of the tissues had tumour cellularities between 10% and 20%. The vast majority (72%) of the specimens, however, contained mostly tumour cells.

In summary, we provide for the first time quantitative evidence that p53 accumulation in breast tumour tissue is associated with markers of increased cellular proliferation and with the expression of other growth-related proteins, all of which may indicate unfavourable prognosis. We also demonstrate the utility of an ELISA-type immunological assay for p53 protein quantification in breast tumour cytosols. As patient follow-up was not possible in this study, determination of the prognostic usefulness of immunofluorometrically measured p53 protein in breast cancer must await further studies. Furthermore, we have demonstrated that PSA, a new favourable prognostic indicator in breast cancer, is closely associated with other favourable prognostic markers. We expect that our data may have utility in the design and selection of prognostic panels in breast cancer based on quantitative measurements in a single tumour extract.

**Acknowledgements**
This study was supported by a grant to EP Diamandis from the Cancer Research Society Inc., Montreal, PQ, Canada.

**References**

ALLRED DC, CLARK GM, ELLEDGE R, FUQUA SA, BROWN RW, CHAMNESS GC, OSBORNE CK AND MCGUIRE WL. (1993). Association of p53 protein expression with tumour cell proliferation rate and clinical outcome in node-negative breast cancer. *J. Natl Cancer Inst.*, **85**, 200–206.

ANDERSEN TI, HOLM R, NESLAND JM, HEIMDAL KR, OTTESTAD L AND BORRESEN AL. (1993). Prognostic significance of TP53 alterations in breast carcinoma. *Br. J. Cancer*, **68**, 540–548.

BARBARESCHI M, LEONARDI E, MAURI FA, SERIO G AND PALMA PD. (1992). p53 and c-erbB-2 protein expression in breast carcinomas. An immunohistochemical study including correlations with receptor status, proliferation markers, and clinical stage in human breast cancer. *Am. J. Clin. Pathol.*, **98**, 408–418.

CARTER CL, ALLEN C AND HENSON DE. (1989). Relation of tumour size, lymph node status and survival in 24,740 breast cancer cases. *Cancer*, **63**, 181–187.

CHEVALLIER B, HEINTZMAN F, MOSSERI V, DAUCE JP, BASTIL P, GRAIC Y, BRUNELLE P, BASUYAU JP, COMOZ M AND ASSELAIN B. (1988). Prognostic value of estrogen and progesterone receptor in operable breast cancer. *Cancer*, **62**, 2517–2524.

CHRISTOPOULOS TK AND DIAMANDIS EP. (1992). Enzymatically-amplified time-resolved fluorescence immunoassay with terbium chelates. *Anal. Chem.*, **64**, 342–346.

CONTESSO G, SACCANI JOTTI G AND BONADONNA G. (1989). Tumour grade as a prognostic factor in breast cancer. *Eur. J. Cancer*, **25**, 403–409.

DAVIDOFF AM, HUMPHREY PA, IGLEHART JD AND MARKS JR. (1991). Genetic basis for p53 overexpression in human breast cancer. *Proc. Natl Acad. Sci. USA*, **88**, 5006–5010.

DENG G, CHEN LC, SCHOTT DR, THOR A, BHARGAVA V, LJUNG BM, CHEW K AND SMITH HS. (1994). Loss of heterozygosity and p53 mutations in breast cancer. *Cancer Res.*, **54**, 449–505.

DIAMANDIS EP AND LEVESQUE MA. (1995). Assessment of p53 protein overexpression by non-immunohistochemical methods. *J. Pathol.*, **175**, 93–95.

DIAMANDIS EP, YU H AND SUTHERLAND DJA. (1994). Detection of prostate specific antigen immunoreactivity in breast tumours. *Breast Cancer Res. Treat.*, **32**, 301–310.

DOMAGALA W, MARKIEWSKI M, KUBIAK R, BARTKOWIAK J AND OSBORN M. (1993). Immunohistochemical profile of invasive lobular carcinoma of the breast: predominantly vimentin and p53 protein negative, cathepsin D and oestrogen receptor positive. *Virchows Arch. A. Pathol. Anat. Histopathol.*, **423**, 497–502.

DRESSLER LG, SEAMER LC, OWENS MA, CLARK GM AND MCGUIRE WL. (1988). DNA flow cytometry and prognostic factors in 1131 frozen breast cancer specimens. *Cancer*, **61**, 420–427.

ELLEDGE RM, MCGUIRE WL AND OSBORNE CK. (1992). Prognostic factors in breast cancer. *Semin Oncol.*, **19**, 244–253.

ELLEDGE RM, FUQUA SA, CLARK GM, PUJOL P, ALLRED DC AND MCGUIRE WL. (1993). Prognostic significance of p53 gene alterations in node-negative breast cancer. *Breast Cancer Res. Treat.*, **26**, 225–235.

FAILLE A, DE CREMOUX P, EXTRA JM, LINARES G, ESPIE M, BOURSTYN E, DE ROCQUANCOURT A, GIACCHETTI S, MARLY M AND CALVO F. (1994). p53 mutations and overexpression in locally advanced breast cancers. *Br. J. Cancer*, **69**, 1145–1150.

FISHER B, REDMOND C, FISHER ER AND CAPLAN R. (1988). Relative worth of estrogen or progesterone receptors and pathologic characteristics of differentiation as indicators of prognosis in node-negative breast cancer: findings from the National Surgical Adjuvant Breast and Bowel Protocol B-06. *J. Clin. Oncol.*, **6**, 1076–1987.

FREBOURG T, KASSEL J, LAM KT, GRYKA MA, BARBIER N, ANDERSEN TI, BORRESEN AL AND FRIEND SH. (1992). Germ-line mutations of the p53 tumour suppressor gene in patients with high risk for cancer inactivate the p53 protein. *Proc. Natl Acad. Sci. USA*, **89**, 6413–6417.

FRIEDRICHS K, GLUBA S, EIDTMANN H AND JONAT W. (1993). Overexpression of p53 and prognosis in breast cancer. *Cancer*, **72**, 3641–3647.

GASPARINI G, BORACCHI P, BEVILACQUA P, MEZZETTI M, POZZA F AND WEIDNER N. (1994). A multiparametric study on the prognostic value of epidermal growth factor receptor in operable breast carcinoma. *Breast Cancer Res. Treat*, **29**, 59–71.

GIAI M, ROAGNA R, PONZONE R, DE BORTOLI M, DATI C AND SISMONDI P. (1994). Prognostic and predictive relevance of c-erbB-2 and ras expression in node-positive and node-negative breast cancer. *Anticancer Res.*, **14**, 1441–1450.

GLEBOV OK, MCKENZIE KE, WHITE CA AND SUKUMAR S. (1994). Frequent p53 gene mutations and novel alleles in familial breast cancer. *Cancer Res.*, **54**, 3703–3709.

GNANT MF, BLIJHAM GH, REINER A, SCHEMPER M, REYNDERS M, SCHUTTE B, VAN ASCHE C, STEGER G AND JAKESZ R. (1993). Aneuploidy fraction but not DNA index is important for the prognosis of patients with stage I and II breast cancer–10-year results. *Ann. Oncol.*, **4**, 643–650.

HANZAL E, GITSCH G, KOHLBERGER P, DADAK C, MIECHOWIEKA N AND BREITENECKER G. (1992). Immunohistochemical detection of mutant p53 suppressor gene product in patients with breast cancer: influence on metastasis-free survival. *Anticancer Res.*, **12**, 2325–2329.

HASSAPOGLIDOU S, DIAMANDIS EP AND SUTHERLAND DJA. (1993). Quantification of p53 protein in tumour cell lines, breast tumour tissue extracts and serum with time-resolved immunofluorometry. *Oncogene*, **8**, 1501–1509.

HOLLSTEIN M, SIDRANSKY D, VOGELSTEIN B AND HARRIS CC. (1991). p53 mutations in human cancers. *Science*, **253**, 49–53.

HORAK E, SMITH K, BROMLEY L, LEJEUNE S, GREENALL M, LANE D AND HARRIS AL. (1991). Mutant p53, EGF receptor and c-erbB2 expression in human breast cancer. *Oncogene*, **6**, 2277–2284.

HURLIMANN J, CHAUBERT P AND BENHATTAR J. (1994). p53 gene alterations and p53 protein accumulation in infiltrating ductal breast carcinomas: correlation between immunohistochemical and molecular biology techniques. *Mod. Pathol.*, **7**, 423–428.

ISOLA J, VISAKORPI T, HOLLI K AND KALLIONIEMI OP. (1992). Association of overexpression of tumour suppressor protein p53 with rapid cell proliferation and poor prognosis in node-negative breast cancer patients. *J. Natl Cancer Inst.*, **84**, 1109–1114.

ISOLA J, WEITZ S, VISAKORPI T, HOLLI K, SHEA R, KHABBAZ N AND KALLIONIEMI OP. (1993). Cathepsin D expression detected by immunohistochemistry has independent prognostic value in axillary node-negative breast cancer. *J. Clin. Oncol.*, **11**, 36–43.

KUERBITZ SJ, PLUNKETT BS, WALSH WV AND KASTAN MB. (1992). Wild-type p53 is a cell cycle checkpoint determinant following irradiation. *Proc. Natl Acad. Sci. USA*, **50**, 379–384.

LEVESQUE MA, DIAMANDIS EP, YU H AND SUTHERLAND DJA. (1994). Quantitative analysis of mutant p53 protein in breast tumour cytosols and study of its association with other biochemical prognostic indicators in breast cancer. *Breast Cancer Res. Treat.*, **30**, 179–195.

LEVESQUE M, YU H, D'COSTA M AND DIAMANDIS EP. (1995*a*). Prostate specific antigen expression by various tumors. *J. Clin. Lab. Anal.*, **9**, 123–128.

LEVESQUE MA, KATSAROS D, YU H, ZOLA P, SISMONDI P, GIARDINA G AND DIAMANDIS EP. (1995*b*). Mutant p53 overexpression is associated with poor outcome in patients with well or moderately differentiated ovarian carcinoma. *Cancer*, **75**, 1327–1338.

LEVINE AJ, MOMAND J AND FINLAY CA. (1991). The p53 tumour suppressor gene. *Nature*, **351**, 453–456.

LIPPONEN P, JI H, AALTOMAA S, SYRJANEN S AND SYRJANEN K. (1993). p53 protein expression in breast cancer as related to histopathological characteristics and prognosis. *Int. J. Cancer*, **55**, 51–56.

LOWE SW, SCHMITT EM, SMITH SW, OSBORNE BA AND JACKS T. (1993). p53 is required for radiation induced apoptosis in mouse thymocytes. *Nature*, **362**, 847–849.

LOWRY OH, ROSEBOROUGH NJ, FARR AL AND RANDALL RJ. (1951). Protein measurement with folin-phenol reagent. *J. Biol. Chem.*, **193**, 265–275.

MARCHETTI A, BUTTITTA F, PELLEGRINI S, CAMPANI D, DIELLA F, CECCHETTI D, CALLAHAN R AND BISTOCCHI M. (1993). p53 mutations and histological type in invasive breast carcinoma. *Cancer Res.*, **53**, 4665–4669.

MAZARS R, SPINARDI L, BENCHEIKH M, SIMONY-LAFONTAINE J, JEANTOUR P AND THEILLET C. (1992). p53 mutations occur in aggressive breast cancer. *Cancer Res.*, **52**, 3918–3922.

MEYER JS AND HE W. (1994). High proliferative rates demonstrated by bromodeoxyuridine labeling index in breast carcinomas with p53 overexpression. *J. Surg. Oncol.*, **56**, 146–152.

NATHAN B, GUSTERSON B, JADAYEL D, O'HARE M, ANBAZHAGAN R, JAYATILAKE H, EBBS S, MICKLEM K, PRICE K AND GELBER R. (1994). Expression of BCL-2 in primary breast cancer and its correlation with tumour phenotype. For the International (Ludwig) Breast Cancer Study Group. *Ann. Oncol.*, **5**, 409–414.

NEGRINI M, SABBIONI S, HALDAR S, POSSATI L, CASTAGNOLI A, CORALLINI A, BARBANTI-BRODANO G AND CROCE CM. (1994). Tumour and growth suppression of breast cancer cells by chromosome 17-associated functions. *Cancer Res.*, **54**, 1818–1824.

O'MALLEY FP, VNENCAK-JONES CL, DUPONT WD, PARL F, MANNING S AND PAGE DL. (1994). p53 mutations are confined to the comedo-type ductal carcinoma in situ of the breast. Immunohistochemical and sequencing data. *Lab. Invest.*, **71**, 67–72.

OSTROWSKI JL, SAWAN A, HENRY L, WRIGHT C, HENRY JA, HENNESSEY C, LENNARD TJ, ANGUS B AND HORNE CH. (1991). p53 expression in human breast cancer related to survival and prognostic factors: an immunohistochemical study. *J. Pathol.*, **164**, 75–81.

POLLER DN, HUTCHINGS CE, GALEA M, BELL JA, NICHOLSON RA, ELSTON CW, BLAMEY RW AND ELLIS IO. (1992). p53 protein expression in human breast carcinoma: relationship to expression of epidermal growth factor receptor, c-erbB-2 protein overexpression, and oestrogen receptor. *Br. J. Cancer*, **66**, 583–588.

RADFORD DM, FAIR K, THOMPSON AM, RITTER JH, HOLT M, STEINBRUECK T, WALLACE M, WELLS SA Jr AND DONIS-KELLER HR. (1993). Allelic loss on a chromosome 17 in ductal carcinoma in situ of the breast. *Cancer Res.*, **53**, 2947–2949.

RAILO M, NORDLING S, VON BOGUSLAWSKY K, LEIVONEN M, KYLLONEN L AND VON SMITTEN K. (1993). Prognostic value of Ki-67 immunolabelling in primary operable breast cancer. *Br. J. Cancer*, **68**, 579–583.

SCATCHARD G. (1949). The attraction of proteins for small molecules and ions. *Ann NY Acad. Sci.*, **51**, 660–672.

SCHWARTZ GF, SCHWARTING R, RABINDRANAUTH P AND FINKEL GC. (1993). Clinical applications of serum and tissue markers in malignant disease: breast cancer as the paradigm. *Clin. Chem.*, **39**, 2404–2412.

SHEIKH MS, SHAO ZM, HUSSAIN A AND FONTANA JA. (1993). The p53-binding protein MDM2 is differentially expressed in human breast carcinoma. *Cancer Res.*, **53**, 3226–3228.

SIDRANSKY D, TOKINO T, HELZLSOUER K, ZEHNBAUER B, RAUSCH G, SHELTON B, PRESTIGIACOMO L, VOGELSTEIN B AND DAVIDSON N. (1992). Inherited p53 gene mutations in breast cancer. *Cancer Res.*, **52**, 2984–2986.

SILVESTRINI R, BENINI E, DAIDONE MG, VERONI S, BORACCHI P, CAPELLETTI V, DI FRONZO G AND VERONESI U. (1993). p53 as an independent prognostic marker in lymph node-negative breast cancer patients. *J. Natl Cancer Inst.*, **85**, 965–970.

SINGH S, SIMON M, MEYBOHM I, JANTKE I, JONAT W, MAASS H AND GOEDDE HW. (1993). Human breast cancer: frequent p53 allele loss and protein overexpression. *Hum. Genet.*, **90**, 635–640.

STENMARK-ASKMALM M, STAL O, SULLIVAN S, FERRAUD L, SUN XF, CARSTENSEN J AND NORDENSKJOLD B. (1994). Cellular accumulation of p53 protein: an independent prognostic factor in stage II breast cancer. *Eur. J. Cancer*, **30A**, 175–180.

TAHAN SR, NEUBERG DS, DIEFFENBACH A AND YACOUB L. (1993). Prediction of early relapse and shortened survival in patients with breast cancer by proliferating cell nuclear antigen score. *Cancer*, **71**, 3552–3559.

TANDON AK, CLARK GM, CHAMNESS GC, ULLRICH A AND McGUIRE WL. (1989). HER-2 neu oncogene protein and prognosis in breast cancer. *J. Clin. Oncol.*, **7**, 1120–1128.

THOMPSON AM, HAWKINS RA, ELTON RA, STEEL CM, CHETTY U AND CARTER DC. (1993). pS2 is an independent factor of good prognosis in primary breast cancer. *Br. J. Cancer*, **68**, 93–96.

THOR AD, MOORE DH II, EDGERTON SM, KAWASAKI ES, REIHSAUS E, LYNCH HT, MARCUS JN, SCHWARTZ L, CHEN LC, MAYALL BH AND SMITH HS. (1992). Accumulation of p53 tumour suppressor gene protein: an independent marker of prognosis in breast cancers. *J. Natl Cancer Inst.*, **84**, 845–855.

TOI M, TOMINAGA T, OSAKI A AND TOGE T. (1994). Role of epidermal growth factor receptor expression in primary breast cancer: results of a biochemical study and an immunohistochemical study. *Breast Cancer Res. Treat*, **29**, 51–58.

TSUDA H AND HIROHASHI S. (1994). Association among p53 gene mutation, nuclear accumulation of the p53 protein and aggressive phenotypes in breast cancer. *Int. J. Cancer*, **57**, 498–503.

VOJTESEK B AND LANE DP. (1993). Regulation of p53 protein expression in human breast cancer cell lines. *J. Cell Sci.*, **105**, 607–612.

WITZIG TE, INGLE JN, CHA SS, SCHAID DJ, TABERY RL, WOLD LE, GRANT C, GONCHOROFF NJ AND KATZMANN JA. (1994). DNA ploidy and the percentage of cells in S-phase as prognostic factors for women with lymph node negative breast cancer. *Cancer*, **74**, 1752–1761.

YU H AND DIAMANDIS EP. (1993). Ultrasensitive time-resolved immunofluorometric assay of prostate specific antigen in serum and preliminary clinical studies. *Clin. Chem.*, **39**, 2108–2114.

YU H AND DIAMANDIS EP. (1995). Prostate specific antigen in milk of lactating women. *Clin. Chem.*, **41**, 54–60.

YU H, DIAMANDIS EP, LEVESQUE MA, SISMONDI P, ZOLA P AND KATSAROS D. (1994*a*). Ectopic production of prostate specific antigen by a breast tumor metastatic to the ovary. *J. Clin. Lab. Anal.*, **8**, 251–253.

YU H, DIAMANDIS EP AND SUTHERLAND DJA. (1994*b*). Immunoreactive prostate specific antigen levels in female and male breast tumours and its association with steroid hormone receptors and patient age. *Clin. Biochem.*, **27**, 75–79.

YU H, GIAI M, DIAMANDIS EP, KATSAROS D, SUTHERLAND DJA, LEVESQUE MA, ROAGNA R, PONZONE R AND SISMONDI P. (1995). Prostate specific antigen is a new favourable prognostic indicator for women with breast cancer. *Cancer Res.*, **55**, 2104–2110.